# Tackling change in mental health service delivery: A qualitative evaluation of a lifestyle program targeting mental health staff – Keeping our Staff in Mind (KoSiM)

**DOI:** 10.1002/hpja.633

**Published:** 2022-07-15

**Authors:** Andrew Watkins, Jane Stein‐Parbury, Jackie Curtis, Josephine Poole, Scott Teasdale, Hamish Fibbins, Elisa Rossimel, Oscar Lederman, Philip B. Ward, Simon Rosenbaum, Elizabeth Denney‐Wilson

**Affiliations:** ^1^ Mindgardens Neuroscience Network Sydney NSW Australia; ^2^ Faculty of Health University of Technology Sydney Sydney NSW Australia; ^3^ School of Psychiatry University of New South Wales Sydney Australia; ^4^ Keeping the Body in Mind, South Eastern Sydney Local Health District Sydney NSW Australia; ^5^ Susan Wakil School of Nursing and Midwifery University of Sydney Sydney NSW Australia

**Keywords:** cardiovascular disease, health behaviours, health equity, mental health, workforce development

## Abstract

**Issues addressed:**

People with severe mental illness have adverse health outcomes compared to the general population. Lifestyle interventions are effective in improving health outcomes in this population. Current cultural processes in mental health services do not generally incorporate physical health care practices. Innovative education is required to improve knowledge and confidence of staff in the delivery of preventative health measures.

**Methods:**

The Keeping our Staff in Mind (KoSiM) program delivered a brief lifestyle intervention to mental health staff. A qualitative analysis following the Standards for Reporting Qualitative Research was undertaken. Semi‐structured interviews designed to elicit information about the acceptability of the program and the impact of the intervention on participants' personal and professional lives. The interviews were analysed using thematic analysis, with coding independently developed and reviewed by three authors.

**Results:**

Of the 103 eligible participants, 75 were interviewed. Responses revealed four main themes: (i) positive changes in clinician's approach to physical health care, (ii) improvements in attitudes to self‐care and family wellbeing, (iii) positive changes in workplace culture associated with physical health care delivery and (iv) high levels of acceptability of the program.

**Conclusion:**

The KoSiM model may be useful in other settings as a means of changing the culture of mental health services to better integrate physical health care as a core part of mental health service provision.

**So what?:**

A novel approach using staff focussed lifestyle interventions model may cut through the resistance that is encountered when implementing proven methods of clinical intervention where cultural barriers exist.

## 1 INTRODUCTION

People who experience a severe mental illness (SMI) have a 15‐year reduction in life expectancy compared to the general population.^1^ The majority of deaths are attributable to preventable cardiovascular disease, which are often exacerbated by antipsychotic use.^2^ Lifestyle interventions targeting modifiable risk factors including diet and sedentary behaviour are effective in improving physical health outcomes for people who experience a SMI.^3^, ^4^, ^5^, ^6^ Social stigma, poverty, social isolation, low educational attainment and poor psychological health often lead to lower levels of engagement and follow‐through with general and preventative health services.^7^ This increases the importance of mental health services targeting the physical health of people who experience a SMI through screening and lifestyle interventions.

Whilst mental health professionals are ideally placed to provide lifestyle interventions,^8^ significant barriers exist to delivering this type of care. Diagnostic overshadowing (attributing physical symptoms to a mental health condition), lack of specialist education, low confidence of staff to deliver interventions, heavy workloads are some of the reasons that mental health organisational cultures that do not embrace physical health care.^9^ There have been repeated calls in the literature to increase the amount of physical health training to mental health professionals to improve health care outcomes for those who experience a SMI. Education and training initiatives can have a positive impact on knowledge of physical health issues in SMI, and the confidence and attitudes of mental health professionals to deliver physical health interventions.^10^ However, it has been identified that future research should investigate practice change interventions to increase the delivery of physical health intervention and support.^9^

A healthy workforce is associated with higher levels of staff retention, lower levels of absenteeism, and overall higher rates of productivity amongst health professionals.^11^ However, health professionals often share similar health concerns to the people they treat.^12^ Poor lifestyle habits in clinicians can have a direct impact on the quality of care they provide.^13^ Physical health interventions for staff have been shown to be feasible and acceptable in mental health settings.^14^ Furthermore, some of these interventions have been demonstrated to improve staff morale and their inclination to participate in healthy lifestyle activities.^15^ Furthermore, the promotion of healthy lifestyle behaviours by health professionals is more likely to occur when health professionals themselves engage in healthy lifestyle behaviours.^16^ The physical health of health care professionals themselves is considered important in the acceptance of lifestyle advice, and when health care professionals follow their own advice.^17^

The Keeping the Body in Mind (KBIM) program is a real‐world intervention delivered as part of standard care within a public mental health service that demonstrated that weight gain could be prevented in young people commencing antipsychotic treatment.^18^ Innovations in health care are more likely to become accepted and become widespread in an organisation if shared values exist and are reinforced by influential team members.^19^ To support the scaling‐up of the KBIM program to adults who experience SMI, the Keeping our Staff in Mind (KoSiM) program was implemented prior to its launch, as a facilitator for this health care innovation.^20^ KoSiM provided an intervention to mental health staff that comprised a brief version of the KBIM lifestyle intervention that people with lived experience of SMI received, creating an experiential learning opportunity.^21^ The stated goals of the KoSiM program were improving staff health, instigating culture change regarding the importance of the physical health of people with a lived experience of SMI, and increasing the capability of mental health staff to deliver physical health assessments and interventions. A full description of the KoSiM program and its quantitative outcomes has been published previously.^21^ This report focuses on the qualitative analysis of the KoSiM program, to understand the experiences of staff participants who completed the intervention and comprehend its efficacy and acceptability of its stated goals.

## 2 METHODS

A pragmatic single‐arm intervention study was conducted in a public mental health service, including inpatient and community settings, in Sydney, Australia. Participants received a five‐session individualised lifestyle intervention (delivered over 5 weeks) that incorporated physical activity and nutritional counselling delivered by multidisciplinary teams. An initial assessment was provided to participants which incorporated an exercise physiologist, dietitian and clinical nurse consultant, with a plan formulated. Participants then had access to four weekly sessions with their choice of clinician. The aim of this qualitative study was to investigate the experiences of staff participants including the perceived effect on their own wellbeing as well as the impact on their work practices and families. This qualitative study was initiated and designed by the first author (A.W.). All participants were informed about the study by the interviewer and written informed consent was obtained. The study was approved by the South Eastern Sydney Local Health District Human Research Ethics Committee (HREC 15/054).

The reporting of this research follows the Standards for Reporting Qualitative Research, a list of 21 items that support transparent reporting of qualitative research.^22^ The sampling approach was to collect data from as many participants as possible who met the inclusion criteria in order to reach data adequacy.^23^ Data collection was deemed to be complete when thematic saturation had been reached as determined by three researchers, as indicated by additional data generating minimal or no new knowledge to address the experiences or impact of the intervention on participants.^24^ All staff working in a clinical role who completed the KoSiM program met the inclusion criteria and were offered the opportunity to participate at the end of the final session of the KoSiM program. Consent and baseline data for the KoSiM program was completed by n = 212 people, of whom n = 129 completed the program. Non‐clinical staff (admin, patient service assistants and non‐clinical management) were excluded (n = 26), leaving a total eligible sample of n = 103 participants.

Interviews were conducted by Clinical Nurse Consultants (A.W. – male or J.P. – female) who were clinical leads in the KoSiM program at different sites. The interviews were conducted in person and audio recorded at the completion of the final session of the KoSiM program between April 2015 and September 2016. Brief interviews were conducted lasting between 12 and 33 min in duration and were conducted at the participants usual place of employment. The interviews were conducted using narrative inquiry, a research methodology which captures the perspective of participants constructing a narrative of events considering the relationship between their personal experience and cultural context.^25^, ^26^ The interviews were semi‐structured, open ended, with looping and probing questions used throughout the interviews to garner the specific experiences of participants involvement. Interviews included follow‐up questions and clarifications about their involvement and its influences on their clinical interactions and personal life. The core brief interview questions are contained in Box 1. The interviews were then transcribed by the respective interviewer with identifying information being removed, names were replaced with pseudonyms to assure anonymity.

BOX 1 **Brief interview questions**

How has your experience been with the KoSiM intervention?

What changes (if any) have you made as a result of participating in KoSiM?

Has participating in KoSiM made you give more consideration to the physical health of other people?

Is there anything that you could suggest that would improve the KoSiM program?

Is there anything else you would like to say about the KoSiM program?

Thematic analysis was utilised for analysing the collected data. Thematic analysis provides precision and consistency and is also an exhaustive process.^27^ This approach has been commonly used to examine the impact of lifestyle interventions and provides sufficient detail for the reader to establish the validity and credibility of the process.^28^ The data was processed according to the method set out by the six phases of thematic analysis described by Braun and Clarke.^29^ This six phase approach involves: familiarisation with the data through transcription, generating initial codes, searching for themes, reviewing themes, defining and naming themes and producing the final written output.^29^

The choice of research design, methodology and theoretical framework, were designed to assist with critical reflection during the research process. Among the program designers and implementation clinicians there was a high desire to see the program reflected in a positive light. The research team recognised the potential for bias in in the construction of knowledge from the qualitative data. The data were analysed by three of the authors (A.W., J.S.P. and E.D.W.), to ensure the data was interpreted with rigorous research standards J.S.P. and E.D.W. who are experienced qualitative researchers were independent from the design and implementation of the KoSiM program.

The analysis process commenced with familiarisation by reading the transcribed texts several times. The next step involved creating notes and developing preliminary themes via an inductive process performed collaboratively between researchers. These themes were later developed by using a constant comparison technique.^30^ From this comparison broad themes were created via journaling and meetings between researchers. Initial categories were created and then broadened to develop hierarchies of concepts and themes. A comprehensive list of codes was developed and reviewed, which was used to analyse the data. The authors debriefed throughout the data analysis process, reflecting on understanding and responses to the quotes, and moved back and forth between the phases. The steps used in the analysis were similar to those outlined by Nowell et al,^28^ which aimed to establish rigour and trustworthiness in a thematic analysis. The researchers considered the finalised themes and their supporting quotes, any differences were deliberated on and determined by mutual agreement.

## 3 RESULTS

Of the 103 eligible participants that completed the KoSiM program 75 partook in qualitative interviews about the program. The high level of participation allowed the findings to achieve data adequacy. Participants ranged in age from 24 to 63 years, most participants were female n = 49 (65%). Forty (53%) participants were nurses, 29 (39%) were allied health professionals (psychologist, social worker, occupational therapist) and 6 (8%) were medical staff.

Data analysis revealed four main themes: changes in the approach to working with a lived experience of SMI; changes in attitude to self‐care and family wellbeing; observed changes in work culture; and general experiences of the overall program. These themes and their sub‐themes are detailed in Table 1.

**TABLE 1:** Qualitative themes

Primary themes	Sub‐themes
Changes in the approach to working with consumers This theme details the changes in the way they approached their practice with mental health consumers to incorporate more physical health.	*Increased knowledge: “easier to explain”* Enhanced comprehension of physical health care issues and how to manage them in mental health consumers.
	*Increased confidence: “more assured”* The increased belief that participants had in feeling capable to implement physical health initiatives to mental health consumers.
	*Greater awareness: “difficult for consumers”* Greater recognition of the additional challenges consumers' face in managing their physical health.
	*Role modelling: “practice what you preach”* The importance of participants representing the healthy lifestyle they hope to encourage consumers to achieve.
	*Motivation to change: “not just their mental health”* Explores the changes to practice that clinicians made during the intervention.
Changes in attitude to self‐care and family wellbeing This theme explains the alterations in approach to their own health and that of their family members.	*Self‐care: “refocused me”* The changes that participants made to their own health during the program.
	*Healthy families: “flow‐on effect”* Describes the broader effects of the program on participants families.
Observed changes in work culture This theme explores what changes participants observed in their colleagues and workplace in relation to physical health.	*A healthier workplace: “looking out for each other”* The impact of the program on the workplace ethos around diet and exercise lifestyle.
	*Physical health focus: “a real shift in culture”* Explores the observed changes in workplace practices incorporating physical health care into the clinical service.
General experiences of the overall program	*Overall feedback: “It's been wonderful”* Details the general feedback on the program as a whole.
	*Program criticisms: “if there was a refresher”* Explores how participants felt the program could be improved.

### 3.1 Changes in the approach to working with a lived experience of SMI

Participants expressed that engaging in a wellbeing program for themselves had a flow‐on effect in terms of the way they approached their practice with people with a lived experience of SMI. Participants were able to identify a change in the way they approached people with a lived experience of SMI in relation to their physical health. The changes identified stemmed from multiple sources that included: increased knowledge and confidence in discussing physical health issues, a greater understanding and empathy of the experience of the physical health journey for those with a diagnosis of SMI, role modelling healthy behaviours and motivation for clinicians to make changes to clinical practices around physical health care.

Many participants believed there was an increase in knowledge of physical health care issues and how to manage them. Participants identified a diverse range of areas in which they improved their knowledge base; this included a better understanding of why physical health issues arose with such frequency amongst people who experience SMI, and how both the mental health service, and they as individuals, could help people with a lived experience of SMI to manage their physical health challenges with lifestyle interventions.

*I now know how to have basic conversations about food and exercising with consumers which in the past I got stuck at* (Female, 38).

*I learnt about the healthy plate as a way to demonstrate healthy eating, I have used this to talk to consumers about food and it made it easier to explain and talk about nutrition with them* (Male, 29).

The increase in perceived knowledge resulted in an increase in participant's stated confidence. It was identified that many participants felt better equipped to provide support, education and interventions to people with a diagnosis of SMI around a healthy lifestyle and managing their physical health.

*The program gave me more awareness of lifestyle interventions and I feel far more confident in explaining the importance of physical health, healthy diet and exercise* (Female, 26).

*As a professional, felt like I had much better understanding of what lifestyle interventions I have to offer consumers and felt increased confidence in incorporating these in my practice* (Female, 53).

Taking part in the program gave participants a better insight into how people diagnosed with a SMI might experience physical health screening and interventions. This understanding of people diagnosed with a SMI's experiences and expectations promoted a greater sense of empathy not only about poor physical health outcomes but also the challenges that confront people when they attempt lifestyle change.

*It made me think about how difficult it must be for our clients trying to have success with their goals* (Male, 43).

*I thought it was really useful as an empathy building and understanding experience for what you are asking clients to do. It's really changed how I ask questions and how I frame things, it's increased my sensitivity, around asking questions and being really patient, when discussing lifestyle change* (Female, 32).

Several participants in the program expressed a realisation (or understanding) that it is unreasonable to expect people with a lived experience of SMI to follow advice relating to lifestyle change from a clinician who was not prepared to make these changes themselves. Role modelling behaviour change, demonstrating a healthy lifestyle and promoting a healthy culture in the workplace were identified as important actions towards creating a positive influence on people in that service.

*The health service needs to spend money on things that make staff healthy like this program. In health we have a lot of unhealthy people working. If you want people to believe in changing their lifestyle staff need to be role models* (Female, 61).

*I've learnt the practice what you preach mantra, is really important. I used to just say to consumers that they should exercise more. I now go walking or do other physical activities with them, it sounds small but it's really making a difference to how much motivation the consumers I work with have* (Female, 24).

Participants emphasised that by completing the KoSiM program, they had an increased awareness of physical health challenges experienced by people with a SMI diagnosis. This, in turn, provided motivation to clinicians to start addressing physical health issues. Participants expressed a wide variety of changes that they had made to their practice to incorporate lifestyle interventions. These included supporting people experiencing a SMI by gathering resources that could be shared, monitoring for physical health, liaising with other health professionals such as general practitioners, offering lifestyle advice and support and engaging in healthy lifestyle activities.

*Doing KoSiM prompted me to think about how to ask clients questions around their physical health not just their mental health and to keep it on your radar a little bit more, because when it's on your own personal radar it's also easier to keep it on the radar to check out for your own clients as well* (Female, 52).

*KoSiM has influenced the way I practice. I have been more proactive in promoting exercise, healthy eating and healthy lifestyle choices, to consumers I work with. I'm incorporating it into my general conversations with consumers, I've learnt that it's actually a part of their mental health care* (Male, 25).

### 3.2 Changes in attitude to self‐care and family wellbeing

Participants also noted that there were benefits to their own health. These benefits were an increase in awareness of their own physical health and making lifestyle changes to improve their overall health. These benefits were also reported to flow on to the participants family members in a similar way to the participants. The program created an increased awareness of physical health issues and with this awareness participants started addressing health issues improving their own self‐care and that of their families.

Individual participants stated they made a wide variety of changes to their own self‐care that were attributed the program. These reported changes ranged from making minor adjustments to their dietary intake and exercise levels through to more major changes including overhauling sleep hygiene and quitting smoking. Participants framed their changes to their lifestyle in the context of changing their attitude to their own health and prioritising it.

*I have made some really significant changes during KoSiM, I have decreased my caffeine intake, reduced my intake of simple carbohydrates, I've also regularly increased my step count and started doing resistance exercise for the first time and am really enjoying it* (Female, 48).

*I found the program hugely beneficial, on a personal level it did refocused me about having a healthy lifestyle. It got me involved and in being proactive about what my health goals were, how to improve them and learning new information* (Male, 57).

Participants also noted the impact of their participation in the program on their immediate family members. The benefits of education and behaviour changes influenced partners and children of program participants. This often resulted in culture change within family units to commence healthy lifestyle initiatives to achieve better health.

*I had greater awareness over my diet, and the diet of my kids, and husband. My husband and I joined the gym and are going regularly* (Female, 36).

*I organised for my family to do more walking, we go out each evening after dinner and started bushwalking on weekends. The kids are now asking if each meal is healthy too which is quite a change. The program has definitely had a flow on effect on my family* (Male, 30).

### 3.3 Observed changes in work culture

The KoSiM program was observed to have an impact on the overall culture of the workplace. Participants reported that there were changes to attitudes and activities amongst work colleagues, including a healthier culture among staff members and a greater team focus on addressing physical health with those diagnosed with a SMI in the service.

Program participants witnessed changes in their work colleagues in relation to physical health. Group activities between team members started occurring such as attending the gym after work together, lunchtime walking groups, sharing healthy group lunches and a replacement of unhealthy snacks with healthier options at meetings.

*After KoSiM we have been checking in on each other around our health, we comment on each other's lunches, and ask how they are going with exercise. It has been really good for us bonding as a team. I guess it's a shared thing that we are working on, I think it's been a nice change* (Female, 52).

*Everyone in the office is talking about their nutrition and exercise goals and that makes us even more motivated. No one is buying unhealthy takeaway anymore* (Female, 31).

It was also observed by participants that the culture around discussing the physical health of people with a SMI diagnosis became more prominent. This included seeking advice from colleagues on managing physical health issues in people with whom they worked, physical health issues being discussed at clinical review meetings and the commencement of new groups which target physical health.

*The program encouraged some conversations in the workplace about consumers physical activity, diet, and other healthy lifestyle which is really good, and we have not had before. Physical health care now gets included in peoples care plans far more often* (Female, 62).

*I've noticed the attitude to the physical health of consumers really changed after people did KoSiM. It's been really nice to see clinicians organising new physical activity groups and organising a fruit bowl for the waiting room, there has been a real shift in culture* (Male, 47).

### 3.4 General experiences of the overall program

The overall feedback from participants was very positive and most found the KoSiM program helpful. The program was described as practically‐based and personalised to the individual's need.

*It was a great opportunity that I've really valued, and I looked forward to the sessions I had with the team. It's been really nice just being able to sit here on the recipient end and have those interventions to help me improve my care of consumers and think much more about my own health rather than just thinking about other people's health all the time. I was able to implement a lot of it which is great, and I talk about it to family, friends and colleagues all the time* (Female, 33).

*It's been wonderful, everyone form KoSiM has been very personable and person‐centred. It was really lovely to have all the people that I work with go through the program because the camaraderie amongst my colleagues has been great. I felt valued as a participant and it has provided great education for working with consumers as well. I have really valued receiving this opportunity* (Female, 48).

The criticisms of the program offered by participants were minimal and these were framed in the form of suggested improvements to the program. These improvements mostly related to having the KoSiM program last longer and be available to more staff members. More focused feedback suggested incorporating group exercise and cooking sessions. One participant also suggested it would be helpful to have practical exercise sessions to see if exercises were being performed in the prescribed manner.

*I think it would be really helpful if there was a refresher perhaps at one and 2 months after the interventions and also weekly reminders for people to prompt, for example, are you going to the gym, how's your program been going, what days are you planning to go this week* etcetera*, because I think it would of helped me stay on track more* (Male, 53).

*It would have been great to do some group sessions to work with others. I guess with the diet aspect the one on one has been very useful and I did not require a practical session just learning but I feel it would be beneficial to have practical sessions with the exercise physiologist* (Female, 39).

Qualitative feedback from the KoSiM program did not vary greatly amongst participants based upon identified gender or age group. No discernible difference was observed between these groups in their commentary on changes to clinical care or work culture. However, participants that identified as female were more likely to comment on the impact of the intervention on their family, whilst participants over the age of 45 were more likely to comment on a changing attitude to their own self‐care.

## 4 DISCUSSION

This study describes the experiences of participants in a lifestyle intervention targeting mental health staff (KoSiM). We found that the KoSiM program was very well received by interviewed participants with only limited criticism regarding structure and delivery. Many participants felt valued and appreciated by the organisation because of being able to participate in something “for them,” strengthening the impact of the intervention. The positive data from this study suggested that the program was highly acceptable to participants, who found it beneficial for a multitude of reasons.

Participants felt that they had experienced positive changes in their knowledge, attitudes, and behaviours to their own physical health. This aligns with quantitative results from the KoSiM program that demonstrated significant improvements to dietary and exercise patterns and consequently a reduction in waist circumference of mental health staff that participated.^21^, ^31^, ^32^ This study also found changes in attitude beyond just the individual participant and their own physical health. Participants were more focused on the physical health of their immediate families, their work colleagues, and people experiencing SMI with whom they work. This broader effect increases both the personal and professional value of the interventions to the participants.

Improving the health of staff and their families is an important goal in its own right. However, the benefits also go beyond this and have a direct influence on the quality and credibility of care delivered to those experiencing SMI. There are three pathways for which improved physical health of staff may benefit clinical services. First, health staff who engage in healthy lifestyle activities are more likely to influence positive physical health behaviours in people they work with.^33^ Second, feeling valued by an employer with service offerings like KoSiM is likely to lead to greater staff retention and result in a more experienced and consistent workforce.^34^ Third, reducing health related issues that staff experience improves levels of productivity and reduces absenteeism.^35^

We found that found that KoSiM participants reported improved attitudes, knowledge and confidence regarding lifestyle interventions for people experiencing SMI. These results compliment previously published quantitative results of the parent study using the M‐BACK tool, which demonstrated statistically significant changes across these domains.^21^ The M‐BACK is a validated measure of clinician perceptions of barriers, and their knowledge, attitudes and confidence in regard to delivery of metabolic health intervention to people with SMI.^36^ Participants reported a change in workplace culture that included better integration of physical health care. The primary KoSiM study^21^ found a reduction in perceived barriers to delivering physical health on the M‐BACK scale^36^ despite the intervention not specifically addressing the barriers to care.

The findings of this study indicate that the KoSiM program was an acceptable offering that had multiple impacts in the short‐term of the intervention. To truly explore the longer‐term impact of the intervention of the program, a follow‐up study would be of benefit. However, it is worth postulating what the longer‐term impact of the intervention may be. The quantitative measures from the M‐BACK questionnaire also demonstrated significant changes at 16‐week follow‐up in all domains. Of note all scores were further improved from post measures (although not significantly so).^21^ This quantitatively demonstrates that there was no reduction in the achieved changes of attitudes, confidence and knowledge of metabolic health, and no increase to perceived barriers to implementing metabolic initiatives, 3 months after completing the intervention.

The cultural aspects of an organisation must be purposefully moulded; as they are a vital factor on which improvement focused change such as incorporation of physical health care into mental health services is facilitated.^37^ Short‐term organisational culture change has been observed in this study, via, visible manifestations, shared ways of thinking and deeper shared assumptions. To achieve longer‐term practice change in this area it will require authentic participation from staff to promote the prevailing shape of feeling, thinking, discussing and accomplishing that underpin metabolic health practice.^38^ Maintenance of this culture can not exist without strong leadership from the wider organisation to continue supporting metabolic health care as a priority.^39^

Improved clinical practices such as implementing physical health care within mental health services are essential to meet the shifting demands of health care, however, implementing these initiatives can be challenging. The sheer number of changes and the pace of change lead to what is often referred to as change fatigue.^40^ The KoSiM program offers a novel approach for achieving change through offering a service to the staff ultimately tasked with delivering screening and intervention of a similar initiative to people they are working with. Training staff through this approach may achieve greater “buy‐in” in a shorter time frame for what is a priority area of mental health reform.^41^

## 5 LIMITATIONS

Data for this study was collected in 2015 and 2016 within a week of each participant completing their five‐week intervention. Ideally, this data would have been published in a timelier manner, however, it was held back awaiting publication of the primary quantitative article and then delays from the COVID pandemic. Despite the publication delay the data was analysed within 6 months of the completion of data collection, and we believe remains of high relevance.

This study was subject to several methodological limitations. Although the primary aim was to understand the experiences of participants who completed the program, there are aspects to the KoSiM program that cannot be explored by only interviewing those who successfully completed the intervention. The study missed the opportunity to understand why participants may have dropped out of the intervention or why they chose to not take up the opportunity to participate. To draw conclusions as to the success of the program without considering those who dropped out is unwarranted. By drawing a sample group that caters for maximum variation, it would provide a fairer representation of the entire local mental health workforce.^42^ This information could provide further insights as to whether the KoSiM program is a viable method of implementing physical health care in mental health services more generally.

Another limitation is the potential for bias as interviewers were members of the intervention team. This can lead to problems in two ways, first, participants may feel more inclined to tell the interviewer what they want to hear rather than their true reflections on the program. Secondly, the interviewer is a co‐creator of the data and as such could introduce bias into the questioning process.^43^ Interviewers helped mitigate the issue of instigator bias by using a reflective process and carefully structured non‐leading questions.

## 6 CONCLUSION

The current study enhances the understanding of offering an individualised lifestyle program to mental health staff members. The qualitative data explored in this study are indicative that a staff‐focused lifestyle intervention was a beneficial and acceptable form of intervention and training for mental health staff. The program was effective in creating positive change, including staff members initiating lifestyle changes for themselves and increasing their focus on physical health care when working with people with a SMI. The KoSiM model may be implemented in other settings as a means of changing the culture of mental health services to incorporate physical health care as a core part of mental health service provision.

## CONFLICT OF INTEREST

The authors declare no conflict of interest.

## References

[1] Hjorthøj C , Stürup AE , McGrath JJ , Nordentoft M . Years of potential life lost and life expectancy in schizophrenia: a systematic review and meta‐analysis. Lancet Psychiatry. 2017;4(4):295–301. 28237639 10.1016/S2215-0366(17)30078-0

[2] Vancampfort D , Stubbs B , Mitchell AJ , De Hert M , Wampers M , Ward PB , et al. Risk of metabolic syndrome and its components in people with schizophrenia and related psychotic disorders, bipolar disorder and major depressive disorder: a systematic review and meta‐analysis. World Psychiatry. 2015;14(3):339–47. 26407790 10.1002/wps.20252 PMC4592657

[3] Teasdale SB , Ward PB , Rosenbaum S , Samaras K , Stubbs B . Solving a weighty problem: systematic review and meta‐analysis of nutrition interventions in severe mental illness. Br J Psychiatry. 2017;210(2):110–8. 27810893 10.1192/bjp.bp.115.177139

[4] Firth J , Cotter J , Elliott R , French P , Yung AR . A systematic review and meta‐analysis of exercise interventions in schizophrenia patients. Psychol Med. 2015;45(7):1343–61. 25650668 10.1017/S0033291714003110

[5] Matcham F , McNally L , Vogt F . A pilot randomized controlled trial to increase smoking cessation by maintaining National Health Service Stop Smoking Service attendance. Br J Health Psychol. 2014;19(4):795–809. 24289715 10.1111/bjhp.12078

[6] Arango C , Díaz‐Caneja CM , McGorry PD , Rapoport J , Sommer IE , Vorstman JA , et al. Preventive strategies for mental health. Lancet Psychiatry. 2018;5(7):591–604. 29773478 10.1016/S2215-0366(18)30057-9

[7] Stewart‐Brown S , Samaraweera PC , Taggart F , Kandala N‐B , Stranges S . Socioeconomic gradients and mental health: implications for public health. Br J Psychiatry. 2015;206(6):461–5. 25792696 10.1192/bjp.bp.114.147280

[8] Ross A , Bevans M , Brooks AT , Gibbons S , Wallen GR . Nurses and health‐promoting behaviors: knowledge may not translate into self‐care. AORN J. 2017;105(3):267–75. 28241948 10.1016/j.aorn.2016.12.018 PMC5536335

[9] Bailey JM , Bartlem KM , Wiggers JH , Wye PM , Stockings EA , Hodder RK , et al. Systematic review and meta‐analysis of the provision of preventive care for modifiable chronic disease risk behaviours by mental health services. Prev Med Rep. 2019;16:100969. 31497500 10.1016/j.pmedr.2019.100969 PMC6718945

[10] Watkins A , Stein‐Parbury J , Denney‐Wilson E , Ward PB , Rosenbaum S . Upskilling mental health nurses to address the burden of poor metabolic health: a mixed method evaluation. Issues Ment Health Nurs. 2020;41:925–31. 32552212 10.1080/01612840.2020.1744204

[11] Owusu‐Sekyere F . Assessing the effect of physical activity and exercise on nurses' well‐being. Nurs Stand. 2020;35(4):45–50. 10.7748/ns.2020.e11533 32216247

[12] Perry L , Gallagher R , Duffield C . The health and health behaviours of Australian metropolitan nurses: an exploratory study. BMC Nurs. 2015;14:45. 26339200 10.1186/s12912-015-0091-9 PMC4558723

[13] Crisford P , Winzenberg T , Venn A , Schultz M , Aitken D , Cleland V . Factors associated with physical activity promotion by allied and other non‐medical health professionals: a systematic review. Patient Educ Couns. 2018;101(10):1775–85. 29793786 10.1016/j.pec.2018.05.011

[14] Hjorth P , Davidsen AS , Kilian R , Jensen SO , Munk‐Jørgensen P . Intervention to promote physical health in staff within mental health facilities and the impact on patients' physical health. Nord J Psychiatry. 2016;70(1):62–71. 26086689 10.3109/08039488.2015.1050452

[15] Fibbins H , Ward PB , Watkins A , Curtis J , Rosenbaum S . Improving the health of mental health staff through exercise interventions: a systematic review. J Ment Health. 2018;27(2):184–91. 29447044 10.1080/09638237.2018.1437614

[16] Fie S , Norman IJ , While AE . The relationship between physicians' and nurses' personal physical activity habits and their health‐promotion practice: a systematic review. Health Educ J. 2013;72(1):102–19.

[17] Carlier IV , Lamberts RD , Gersons BP . Risk factors for posttraumatic stress symptomatology in police officers: a prospective analysis. J Nerv Ment Dis. 1997;185(8):498–506. 9284863 10.1097/00005053-199708000-00004

[18] Curtis J , Watkins A , Rosenbaum S , Teasdale S , Kalucy M , Samaras K , et al. Evaluating an individualized lifestyle and life skills intervention to prevent antipsychotic‐induced weight gain in first‐episode psychosis. Early Interv Psychiatry. 2016;10(3):267–76. 25721464 10.1111/eip.12230

[19] Barnett J , Vasileiou K , Djemil F , Brooks L , Young T . Understanding innovators' experiences of barriers and facilitators in implementation and diffusion of healthcare service innovations: a qualitative study. BMC Health Serv Res. 2011;11:342. 22176739 10.1186/1472-6963-11-342 PMC3265424

[20] Rosenbaum S , Watkins A , Ward PB , Pearce D , Fitzpatrick K , Curtis J , et al. Psychiatry HeAL thyself! Aust N Z J Psychiatry. 2016;50(6):600. 10.1177/0004867415620023 26619896

[21] Rosenbaum S , Ward PB , Baldeo R , Fibbins H , Jarman R , Lederman O , et al. Changing health workforce attitudes to promote improved physical health in mental health service users: Keeping our Staff in Mind (KoSiM). Health Promot J Austr. 2020;31:447–55. 31925974 10.1002/hpja.320

[22] O'Brien BC , Harris IB , Beckman TJ , Reed DA , Cook DA . Standards for reporting qualitative research: a synthesis of recommendations. Acad Med. 2014;89(9):1245–51. 24979285 10.1097/ACM.0000000000000388

[23] Vasileiou K , Barnett J , Thorpe S , Young T . Characterising and justifying sample size sufficiency in interview‐based studies: systematic analysis of qualitative health research over a 15‐year period. BMC Med Res Methodol. 2018;18(1):148. 30463515 10.1186/s12874-018-0594-7 PMC6249736

[24] Guest G , Namey E , Chen M . A simple method to assess and report thematic saturation in qualitative research. PloS One. 2020;15(5):e0232076. 32369511 10.1371/journal.pone.0232076 PMC7200005

[25] Clandinin DJ , Connelly FM . Narrative inquiry: experience and story in qualitative research. San Francisco, CA: John Wiley & Sons; 2004.

[26] Giacomini MJ . Theory matters in qualitative health research. In: Bourgeault I , Dingwall R , de Vries R , editors. The SAGE handbook of qualitative methods in health research. London, UK: SAGE; 2010. p. 125–56.

[27] King N . Using templates in the thematic analysis of text. In: Cassell C , Symon G , editors. Essential guide to qualitative methods in organizational research. London: SAGE; 2004. p. 256–70.

[28] Nowell LS , Norris JM , White DE , Moules NJ . Thematic analysis: striving to meet the trustworthiness criteria. Int J Qual Methods. 2017;16(1):1609406917733847.

[29] Braun V , Clarke V . Using thematic analysis in psychology. Qual Res Psychol. 2006;3(2):77–101.

[30] Boeije HJ . A purposeful approach to the constant comparative method in the analysis of qualitative interviews. Quality Quantity. 2002;36(4):391–409.

[31] Fibbins H , Ward PB , Curtis J , Watkins A , Lederman O , Morell R , et al. Effectiveness of a brief lifestyle intervention targeting mental health staff: analysis of physical fitness and activity in the Keeping our Staff in Mind study. BMJ Open Sport Exerc Med. 2020;6(1):e000761. 10.1136/bmjsem-2020-000761 PMC7359059 32685186

[32] Rossimel E , Teasdale SB , Poole J , Fibbins H , Curtis J , Watkins A , et al. Keeping our Staff in Mind: dietary results of a lifestyle intervention targeting mental health staff. Health Promot J Austr. 2021;32(3):451–7. 32589312 10.1002/hpja.377

[33] Esposito EM , Fitzpatrick JJ . Registered nurses' beliefs of the benefits of exercise, their exercise behaviour and their patient teaching regarding exercise. Int J Nurs Pract. 2011;17(4):351–6. 21781214 10.1111/j.1440-172X.2011.01951.x

[34] Keyworth C , Epton T , Goldthorpe J , Calam R , Armitage CJ . Delivering opportunistic behavior change interventions: a systematic review of systematic reviews. Prev Sci. 2020;21(3):319–31. 32067156 10.1007/s11121-020-01087-6 PMC7056685

[35] Letvak S , Ruhm C , Gupta S . Differences in health, productivity and quality of care in younger and older nurses. J Nurs Manag. 2013;21(7):914–21. 24112420 10.1111/jonm.12181

[36] Watkins A , Rosenbaum S , Ward PB , Patching J , Denney‐Wilson E , Stein‐Parbury J . The validity and reliability characteristics of the M‐BACK questionnaire to assess the barriers, attitudes, confidence, and knowledge of mental health staff regarding metabolic health of mental health service users. Front Public Health. 2017;5:321. 29312914 10.3389/fpubh.2017.00321 PMC5732257

[37] Braithwaite J , Herkes J , Ludlow K , Testa L , Lamprell G . Association between organisational and workplace cultures, and patient outcomes: systematic review. BMJ Open. 2017;7(11):e017708. 10.1136/bmjopen-2017-017708 PMC5695304 29122796

[38] Mannion R , Davies H . Understanding organisational culture for healthcare quality improvement. BMJ. 2018;363:k4907. 30487286 10.1136/bmj.k4907 PMC6260242

[39] Bradley EH , Brewster AL , McNatt Z , Linnander EL , Cherlin E , Fosburgh H , et al. How guiding coalitions promote positive culture change in hospitals: a longitudinal mixed methods interventional study. BMJ Qual Saf. 2018;27(3):218–25. 10.1136/bmjqs-2017-006574 PMC5867433 29101290

[40] McMillan K , Perron A . Nurses amidst change: the concept of change fatigue offers an alternative perspective on organizational change. Policy Polit Nurs Pract. 2013;14(1):26–32. 23549748 10.1177/1527154413481811

[41] National Mental Health Commission . Equally well consensus statement: improving the physical health and wellbeing of people living with mental illness in Australia. Sydney: NMHC; 2016.

[42] Seidman I . Interviewing as qualitative research: a guide for researchers in education and the social sciences. New York, NY: Teachers College Press; 2006.

[43] McGrath C , Palmgren PJ , Liljedahl M . Twelve tips for conducting qualitative research interviews. Med Teach. 2019;41(9):1002–6. 30261797 10.1080/0142159X.2018.1497149

